# Promoting Wellness Through Mobile Health Technology in a College Student Population: Protocol Development and Pilot Study

**DOI:** 10.2196/16474

**Published:** 2020-04-03

**Authors:** Susanne B Haga, Ryan Shaw, Charles Kneifel, Sarah J Bond, Geoffrey S Ginsburg

**Affiliations:** 1 Center for Applied Genomics & Precision Medicine Department of Medicine Duke University School of Medicine Durham, NC United States; 2 School of Nursing Duke University Durham, NC United States; 3 Office of Information Technology Duke University Durham, NC United States; 4 Duke University Durham, NC United States

**Keywords:** college students, wearables, wellness

## Abstract

**Background:**

The health and well-being of college students has garnered widespread attention and concern in recent years. At the same time, the expansion and evaluation of digital technologies has grown in recent years for different target populations.

**Objective:**

This protocol aims to describe a pilot feasibility study on wearables to assess student interest and to gather baseline data from college freshmen, for the academic year 2019 to 2020.

**Methods:**

All full-time college freshmen residing in a single residence hall were eligible to participate. Study invitations were sent by post and email 5 weeks prior to move-in. Web-based enrollment and in-person attendance at study orientation sessions were mandatory. We provided the incoming freshmen with a wearable and study app. Wearable data and weekly survey data will be collected through the study app and analyzed. We have collected demographic, enrollment, and attrition data and the number and type of support requests from students.

**Results:**

The planning phase of the WearDuke initiative was completed in 2018 to 2019, and the pilot study was launched in July 2019. Of the 175 students invited, 120 enrolled and 114 started the study; 107 students remained active participants till the end of the fall semester. For Apple Watch participants (the majority of study population), weekly survey completion rates ranged from 70% (74/106) to 96% (95/99).

**Conclusions:**

Halfway through the pilot, we noticed that the initiative has been received positively by the students with minimal attrition. The short- and long-term benefits may be substantial for students, the campus, the utilization of health services, and long-term health.

**International Registered Report Identifier (IRRID):**

DERR1-10.2196/16474

## Introduction

### Background

The transition to college can be an emotionally challenging time, with new experiences, pressures, choices, and independence [1-5]. Colleges and universities have developed a wide range of resources to support student health, well-being, and academic success. Yet, while these efforts are focused on students’ college years, there remain unmet needs, eg, methods that enable students to monitor health-related behaviors, an awareness of the consequences of unhealthy behaviors, and the establishment of healthy behaviors for the future. Many students have a mobile phone (over 96% of adults aged 18-29 years have reported owning one [6]). Thus, apps and tethered devices can serve as useful tools to track daily habits and health-related variables. These data and tools can help identify trends over the week or a semester and utilize reminders, sleep goals, and other types of alerts to prompt behavior change. Although tracking behavior change through tools, such as apps, has been linked to improved well-being [7,8], there is limited research on the use of wearable technologies for improving well-being, particularly among college students who are transitioning to adulthood. To this end, we are launching a scalable, hybrid population health, research, and educational initiative focused on an undergraduate student population. The goals of the initiative are to promote health awareness and engagement, establish individual healthy behaviors, promote a campus culture that fosters healthy living, and provide unique student-centered research and learning opportunities.

Our initiative in promoting student health and wellness capitalizes on 2 major disciplines: (1) population health and (2) digital technologies. Population health has become a central focus of research and an overarching goal for health systems and communities [9]. This focus on population health does not preclude individual-based interventions, rather, the two are inextricably linked. Many factors affect population health, including social determinants, the physical environment, the workplace, access to care, and behaviors. On a population level, greater effort is needed for more comprehensive assessments for risk stratification and to advance the understanding of effective interventions for particular populations.

Emerging digital or mobile health (mHealth) technologies such as smartphone apps, sensors, and wearables and connected devices can collect health-related data from individuals and populations continuously in their daily environment and analyze that data, thus creating a feedback loop to deliver in-time intervention(s) that allow people to self-manage their health and make better choices [10].Furthermore, these data can provide clinicians with a more complete picture of patient health to enable more informed and precise treatment decision making [11]. Owing to the ubiquity of smartphones, the use of mHealth technologies to measure and record health-related behaviors and clinical parameters has become increasingly convenient and useful for research [12]. These tools can increase access to research opportunities across diverse populations, collect near real-time data, and reduce costs for population health research by forgoing in-person visits for assessment and reducing study staff personnel. For example, the national *All of Us* Research Program (formerly Precision Medicine Initiative) recently selected Fitbit to pilot in up to 10,000 participants to record heart rate, physical activity, and sleep.

In addition to sensors embedded in a mobile phone, there are many connected devices that tether to smartphones that allow for the collection of health-related data, eg, glucometers, wrist-worn accelerometers, wireless scales, and portable electrocardiograms. Activity trackers are some of the most popular connected devices and include Fitbit, GENEactiv, Polar, Apple Watch, Vivosmart (Garmin), and Verily Study Watch, among others. Many of these devices also track sleep by largely relying on actigraphy and utilize an accelerometer-based measurement algorithm to estimate total sleep time. Many apps can also record sleep patterns by monitoring movement using a smartphone placed on the bed and, similar to wearables, monitor sleep through a microphone and acoustics. Other devices can be placed under the mattress or at the bedside to monitor pressure, movement, or sound.

### Overall Aim

Several studies have shown promising evidence that support the feasibility, acceptability, and limited effectiveness of digital interventions for behavior change [13-19]. However, many of these studies have been conducted for short durations and on small sample sizes [13]. Through the use of wearable devices, this initiative aims to increase students’ awareness about the importance of activity and good sleep habits through self-tracking and to develop and provide interventions and tools to help achieve healthy behaviors during and following college. We are currently conducting the first of 2 pilot studies to assess student interest, adherence, feasibility, and staffing needs and to gather baseline data through wearables and surveys on stress, diet, physical activity, and sleep behaviors. The pilot data will inform the larger launch of interventions tailored per student behaviors for the entire freshman class.

## Methods

### Design

Although there are many types of behaviors to target, we initially focused on activity and sleep, as poor sleep habits have a profound waterfall effect on not only health but also on academic and social elements [20-22]. Poor quantity and quality of sleep can affect individuals of all ages, gender, and racial and ethnic backgrounds. Many adults fall short of the recommended goal of 7 or more hours of daily sleep [23], with approximately 35% of US adults reporting insufficient sleep [24,25]. In college students, higher rates of insufficient sleep have been reported, with one study reporting 70% of students with inadequate sleep [26,27].

To our knowledge, this type of an initiative has not been implemented in the United States in a campus-wide setting. Preparing for such a large endeavor involved discussions and collaborations with 3 major groups on campus: (1) university leadership and administrators, (2) information technology staff, and (3) students. Given the focus on students, support from the university administration was critical to the development and implementation of such a large initiative. We convened discussions with campus administrators in student health, student wellness, and student affairs to identify support and begin to outline the initiative and develop a proposal. With their commitment, we applied and received funding from the Office of the Provost.

We proposed a 3-year plan to develop and launch this initiative to an entire freshman class, which has been described in detail in the following sections. Specifically, the 3-year proposal included a year-long planning process, followed by 2 year-long pilot studies to assess the feasibility and acceptability of wearables and the impact of connecting students to campus interventions to promote healthy behaviors.

### Year 1: Planning Phase

During the planning phase, we assembled an interdisciplinary faculty advisory team with expertise in mHealth technologies, app development, health behaviors, data science, psychology, sleep disorders, and student affairs and student health with continued participation from campus administrators. The faculty team advised the principal investigators on the development, infrastructure, and implementation of the initiative and is being briefed monthly. To successfully develop and implement an initiative intended for students, we considered it essential to involve students at every stage of planning, development, and implementation. Thus, in parallel with establishing a faculty advisory team, we established a student advisory committee. The student team was responsible for gathering feedback from students about their interest and concerns about the initiative; leading the selection of the name, logo design, and wearable; informing the development of an iOS app; creating the website; and outlining the protocol for the initiative (the student team was supported by the Duke Bass Connections program).

#### Information Technology Solution

With the proposed use of digital technologies and the collection of multiple types of data, we engaged with the Duke’s Office of Information Technology (OIT) staff to develop a strategy for managing the full lifecycle of app development, deployment, and data collection processes. Our team worked very closely with software developers from the campus to develop the infrastructure for data collection, analysis, secure data storage, and technical support for the students. This included the development of a study app and website as well. Furthermore, the OIT staff are instrumental in working with the institutional review board and security office to ensure that the study meets all security and regulatory requirements.

**Figure 1:**
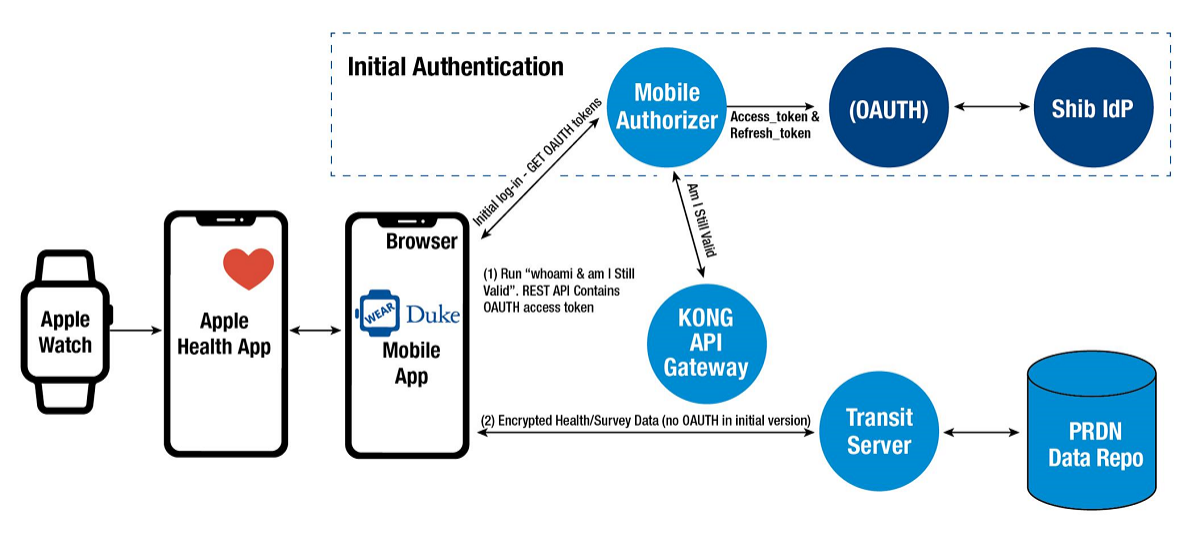
WearDuke iOS architecture. OAUTH: open authorization; Shib IdP: Shibboleth identity provider; KONG: Kong API Gateway; API: Application programming interface; REST API: Representational state transfer API; PRDN: Protected research data network; Repo: Repository; App: Application.

To receive data from Apple Watch (Apple Inc) and push weekly surveys to participants, we developed a study app with OIT. For the pilot studies, we only developed an iPhone Operating System (iOS) app given that a large proportion of incoming students were estimated to have Apple iPhones based on the current student body data (>90%). This included creating a secure server architecture that allowed us to retrieve and store data for analyses (Figure 1). Access to the WearDuke app through the University App Store was restricted to students who finished the consent process. When installing the WearDuke app, students were asked to allow the app to access each category of study data stored in Apple HealthKit (eg, heart rate, steps). HealthKit is an in-built app that aggregates health-related data on the iPhone. Data from Apple Watch is transferred and stored on HealthKit. The WearDuke app retrieves and encrypts the permitted data from HealthKit and then transfers the encrypted data to a secure server on Duke’s protected network. Any third-party app or any other device connected to the phone that is providing data to the HealthKit will be available to the WearDuke app and collected and stored with the study data. The source of the data (Apple Watch, app, or other device) is included with the data collected. Survey data are also collected in the WearDuke app and encrypted and transferred in the same manner as the data from Apple HealthKit.

#### Student Focus Groups

An undergraduate student team conducted a series of focus groups with undergraduate students to ascertain the general interest about the initiative, the name and logo, the familiarity with wearables, the features of a companion app, the likelihood to complete surveys and wear wearables, and an incentive system. We will continue to have undergraduates involved in the initiative throughout the pilot year to help guide and prepare for the second pilot study.

#### Wearable Selection

For this initiative, we will provide the enrolled students with a choice of wearable to monitor activity and heart rate; sleep behavior data will be gathered through an app. With our student team, we reviewed available wearables based on several criteria: type (wrist or ring), battery life, measurements, style, other features of interest to students, and methods of data access. We were unable to identify wearables that are compatible with both Android and iOS smartphones, have a comprehensive set of desired measures (sleep and activity) that are clinically accurate, and possess other features that are attractive to a college student population. Thus, we decided to offer the Fitbit Charge 3 and Apple Watch 3 to Android and iOS smartphone users for the first pilot study, respectively. We will re-evaluate the choice of wearables for the second pilot, informed by the first pilot study and newly introduced wearable models.

### Year 2: Pilot Study 1

In the pilot feasibility study, we worked with the campus institutional review board, information security and privacy offices, and information technology staff to create an informed consent process that would be both understandable and transparent to young adults. We worked with Duke Web Services in OIT to create a university-approved website for the study.

#### Recruitment and Enrollment

In July 2019, we invited students from a single residence hall (N=175) to participate in the study to assess the feasibility and acceptance of wearables to measure health behavior and to identify trends in health behaviors over the students’ freshman year. To be eligible for the study, students must be enrolled as a full-time freshman (Class of 2023), residing in the selected residence hall approved for the study; college freshmen residing in other dorms were not eligible to participate. Students received a letter in the post and by email about the study, inviting them to enroll after reviewing the website; adult-aged students interested in enrolling completed an electronic informed consent form. Students must acknowledge reviewing each section of the electronic consent form (in the checkbox at the end of each section) and sign the form. For students aged under 18 years, parental consent was obtained first, followed by student informed assent (identical in content to the student consent). We worked with OIT to develop a process using institutional data to ensure that only students who met the eligibility criteria were able to be included in the study—a solution using group management that includes information about students who turn 18 after enrolling (with parental consent) to ensure that we are collecting data from properly consented enrollees. Participants were required to attend an in-person orientation the week before the fall semester began; study investigators described the study, benefits, and risks before distributing the wearables. Assistance was provided as needed to set up the wearable and connect to their phones and install and set up the app. OIT support was available throughout the study to address student issues with the wearable or companion app. A WearDuke email was established to facilitate communication with the students.

#### Pilot Study 1: Methodology and Measures

For the first pilot study, we are primarily interested in ascertaining feasibility, student interest, and student experiences as well as infrastructure needs. Feasibility will be measured with study records and feedback regarding the number enrolled, number of withdrawals, frequency of wearing the wearable daily, survey completion rate, and use of information technology support services. In addition to the data on wearables (Table 1), we will administer weekly surveys to gather more information about students’ sleep habits, caffeine use, overall health and mental health, and academic performance (Table 2). We administered a baseline/demographic survey following enrollment. Students with an iOS smartphone were asked to install the developed companion app to complete surveys. Students with Android-based smartphones use the Fitbit app and will complete surveys through Research Electronic Data Capture (REDCap), an electronic data capture tool hosted at Duke University [28,29]. REDCap is a secure, web-based software platform designed to support data capture for research studies. All pilot data will be stored on internal secure servers within the university. We will assign each participating student a unique study identification number. We will review and apply what we learned from year 1 and begin to develop intervention components for year 2.

To remain enrolled in the study, students must maintain an active participation status in the study through the completion of half of the surveys (2 per month) and wearing the watch a minimum of 3 days weekly. Adherence is determined through the analysis of heart rate data from the raw wearable data; an hour worn is defined by collecting at least one heart rate sample from the wearable within that hour. Thus, the *set* of hours of all heart rate samples within a day is defined to be the number of hours the user has worn the watch for that day (ie, the hours in which the samples occurred are 0, 2, 2, 13, 13, 14, 15, which is 5 hours worn). The student must wear the watch for 8 hours to be counted as worn. Incentives were provided to students who completed weekly surveys and wore the wearable for at least the minimum number of days weekly.

**Table 1:** Mobile device data collection.

Data type	Fitbit Charge 3	Apple Watch 3
Activity/exercise	Daily: steps taken, distance, and floors climbed; minutes lightly, fairly, and very active; minutes sedentary	Daily: steps, distance, flights climbed, exercise time, and stand hours
Energy	Daily: calories burned	Daily: basal and active energy burned
Heart rate	Daily average: heart rate and heart rate zone	Daily average: resting heart rate, walking heart rate, and heart rate variability
Sleep analysis	Daily: time asleep and sleep stages (Rapid eye movement, light, and deep)	Native Apple app (clock) or other third-party app (student’s choice)

**Table 2:** Weekly surveys.

Subject/topic	Survey instrument
Demographics	Race, gender, and campus activities
Sleep (habits, environment, and circadian preference)	Sleep/wake behavior problems Use of sleep aides (medications, music, and blinders; adapted from National Sleep Foundation [30]) Sleep quality (Patient-Reported Outcomes Measurement Survey (PROMIS) Sleep Practices and Sleep Disturbance) and daytime sleepiness (PROMIS SRI) [31,32] Circadian preference (Morningness-Eveningness Reduced Questionnaire [33]) Physical environment/number of roommates
Mental health	Depression (Center for Epidemiologic Studies Depression scale) [34] Stress (Cohen’s Perceived Stress Scale [35])
Nutrition (habits and knowledge)	Nutrition knowledge and habits (adapted from [36]) Caffeine intake [37]
General health status	General physical/mental health status [38] Reported number of absences owing to sickness Reported number of visits to student health
Academic schedule/performance	Intended major/minor Courses/schedule Academic performance (GPA) Average hours per week for campus activities, work study, and/or employment
Physical activity	Physical activity habits (adapted from [36]) Participation in campus programs
Experience with wearables and apps	Use of sleep tools/alerts/tracking Experience with wearables and health-related apps

### Year 3: Pilot Study 2

Data collected from the first pilot will inform an expanded, second pilot study that will focus on the evaluation of interventions to promote healthy behaviors. In this second larger study, we will implement an improved app and incentive strategy, continue to monitor activity and sleep behavior, and evaluate the uptake and effectiveness and use of campus activities and interventions. Our intervention will be guided by theory-driven behavior change principles that leverage capabilities of continuous monitoring technologies and address the unique preference of individuals. For this study, we will use the Healthy Apps 4 M’s conceptual model of monitoring, modeling, motivating, and modifying [39]. These principles will help guide our intervention development that will allow for near real-time interventions based on continually observed behavioral response data.

Educational interventions may include workshops, in-person or web-based guest speakers, and sending out healthy tips via push notifications. Physical activity interventions will be available for students at all levels and experiences, individual and group based. Sleep interventions may include both physical sleep aides (eg, pillows) or guidance for establishing a nightly routine to enable adequate sleep quality and quantity. For example, students may benefit from learning how to de-stress, perhaps through simply reducing mobile phone use before going to sleep (it is estimated that about 40% of Americans, including 72% of adolescents, use mobile technology before going to sleep [40,41]). Ideally, we will alert students to various interventions based on their preferences and wearable data. Assuming successful completion of the pilot studies, we hope to expand the initiative to the entire incoming class.

As with the first pilot study, we will re-assess student interest and experiences through the enrollment rate, drop-out rate, daily wearable adherence, damage to wearables, survey completion rate, and use of information technology support services. In addition to objective data collected from the wearables, we will administer weekly surveys to gather more information about students’ sleep habits, caffeine use, overall health and mental health, and academic performance. The second pilot will introduce app notifications based on student preferences and wearable data for activities, workshops, or other resources to promote well-being.

### Data Analysis

This is an exploratory study, and there are no interventions or hypotheses. As these pilot studies are intended to primarily assess feasibility and acceptance, the project is not powered to detect statistically significant effects. Descriptive statistics and some nonparametric tests, eg, Wilcoxon signed rank tests, Fisher exact tests, and other appropriate measures will be calculated using the R environment. We will assess student variables such as gender and intended major with regard to the survey and wearables data. Depending on the scoring algorithm for each survey instrument, any missing data may prohibit the generation of a score. In other cases, descriptive statistics are generated per question, and “prefer not to answer” is a part of the range of responses quantified. Working with Duke OIT, we can provide our analytics staff with tools such as R-Studio, as well as state of the art Python-based tools for the analysis of very large datasets.

### Educational Opportunities

In addition to promoting healthy lifestyles for students and a healthy campus culture, we envision that students and faculty will have the opportunity to conduct research with anonymized datasets, in turn providing students with a real-world dataset to gain skills in research and data analysis. We hope to have the opportunity to work with students to develop smaller studies within the larger group of WearDuke participants to address or gather data for other areas not currently addressed. Through this experience, we anticipate that they will learn about study design, generating hypotheses, survey development and other research methodology, human subject protections, and the analysis of complex datasets. Students may also work on improvements to the app or the development of new campus interventions to promote healthy behaviors.

The study has also allowed us to work with classes across campus such as computer science and engineering. For example, an Android development class created a prototype for the WearDuke study. Not only did this allow students to learn how to work with clients and develop a product for a real-world project but also it provided a beta version and wireframe of an Android version of the study app. We would then be able to take this student work to professional developers as a prototype that can be built upon. We expect that this educational approach will be repeated, and future classes will build an additional app or technology-related features for the study. This may include testing new digital health technologies that could measure sleep, among others.

## Results

**Figure 2:**
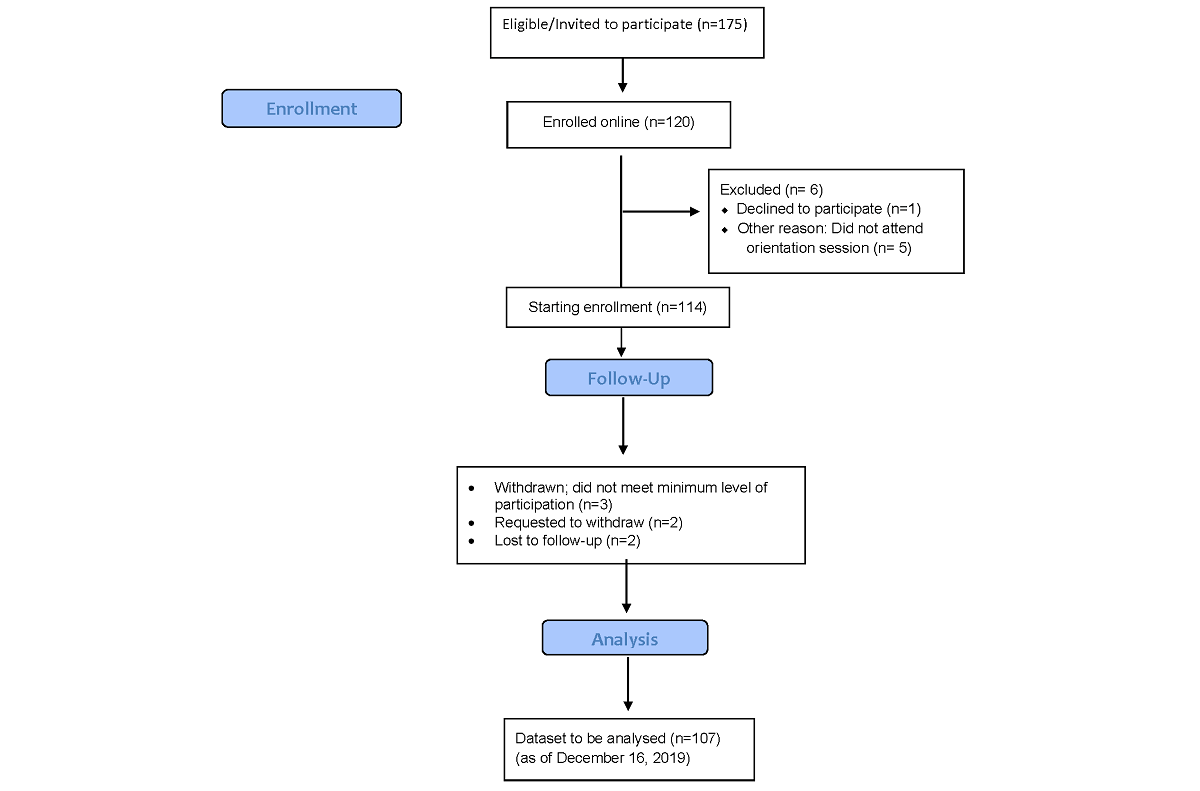
CONSORT Flowchart of WearDuke initiative through end of fall semester.

We completed the year-long planning phase and obtained approval from the Duke University Campus Institutional Review Board in June 2019. The website was made publicly accessible in July 2019 [42]. The first pilot study was launched in July 2019 in a single freshman residence hall. An electronic consent process was established, and all students attended a mandatory orientation session on August 24 to 25, 2019. A total of 175 students were eligible to participate, and 120 (68%) students consented to participate, and 114 (65%) students started the study (see Figure 2). Student demographic data are presented in Table 3.

**Figure 3:**
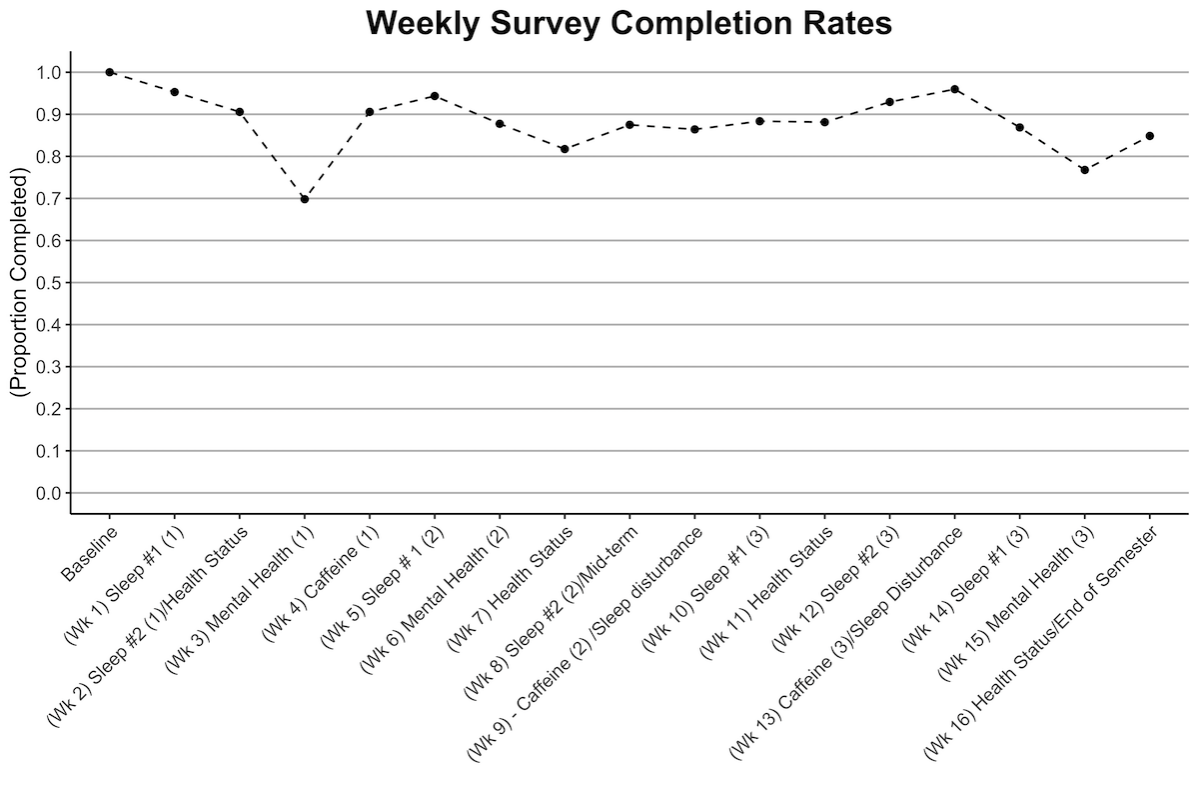
Weekly survey completion rate for Fall 2019 semester for Apple Watch participants.

All students were required to complete a demographic survey during the enrollment process. A survey was administered each week of the semester (16 total surveys). Weekly survey completion rates ranged from a high of 95% to 70% (Figure 3). By the end of the fall semester, a total of 8 students withdrew from the study or were withdrawn by study staff owing to inactive participation. Watch replacements were provided for a total of 10 students for watches that were lost (4), broken (5), or nonfunctional (1). An analysis for each survey and wearables dataset has commenced. In addition to generating summary statistics, we will test for differences between major student demographic features such as gender and intended major for both survey and wearables data as well as test for changes in behaviors across the semester in repeated measures. We aim to publish the initial results of the first pilot study in fall 2020.

**Table 3:** Participant demographic data (N=114).

Characteristics		Values, n (%)
Male		62 (54.3)
**Age (years)**		
	<18	13 (11.4)
	18	95 (83.3)
	19	5 (4.4)
	20 or older	1 (0.88)
Hispanic		15 (13.1)
**Race**		
	White	63 (55.3)
	African American	8 (7.0)
	Asian	27 (23.7)
	Other	2 (1.8)
	More than 1 race	13 (11.4)
**School**		
	Trinity School of Arts & Sciences	85 (74.6)
	Pratt School of Engineering	29 (25.4)
Varsity sports		2 (1.8)
Own an iPhone		106 (93.0)

## Discussion

### Preliminary Findings

In a time of rapid growth of digital technologies in the educational and health space, we seek to promote familiarity with these technologies to improve health and well-being for undergraduate students. No other informal learning opportunity exists for students on campus to gain experience with these technologies, and the potential for new educational opportunities further expands the multiple benefits that this initiative may yield. In the first of the 2 pilot studies commenced, the study launched with 65% of eligible students. Halfway through the study (at the end of the fall semester), the attrition rate was 7%, and the average weekly survey completion rate was 88%.

The transition to college is a period when new and long-term habits are being formed. With ongoing concerns about the health and well-being of college students today, we are developing a tool to help students adjust to the collegiate environment and emphasize the need for students to make time for themselves and establish healthy lifestyles. Such an expansive undertaking requires engagement with many stakeholders and, most importantly, with the targeted student population. We have begun discussions to expand the initiative to the health system and other groups on campus including graduate students, residents/trainees, staff, and faculty. We hope our initial experiences will inform the use of digital technologies in other settings.

We do acknowledge several limitations to this study. The first is the limited sample size of the first pilot, which did not allow us to draw statistical conclusions from the data. The first pilot also does not test the effect of any intervention, so we cannot assess the impact on health and wellness outcomes of using these technologies. Students may become tired or lose enthusiasm to complete the weekly surveys, particularly the repeated surveys, limiting our ability to detect trends over and between semesters. Finally, this study is at a single college campus and does not reflect the diversity of college campuses and student bodies across the United States. We also recognize the economic limitations of broadly implementing an mHealth-based initiative and its general feasibility in other educational settings (or settings with shared living arrangements) or for population health initiatives [43,44].

### Conclusions

College students’ health and well-being has garnered widespread attention and concern in recent years. Similarly, the expansion and evaluation of digital technologies has grown in recent years for different target populations. We believe such an initiative will have both short-term and long-term implications for students in learning about and facilitating healthy behaviors that will optimize well-being and academic performance throughout college as well as establish healthy behaviors that will have a lasting impact on their health and well-being after college. Furthermore, the data and experiences from this initiative can inform the development of similar programs in other educational and group-based settings (eg, military and nursing homes) to improve residents’ overall health in settings that are new/unfamiliar, stressful, and/or resource-limited.

## Abbreviations

mHealth: mobile health
OIT: Office of Information Technology
REDCap: Research Electronic Data Capture
